# The impact of Pleistocene climate change on an ancient arctic–alpine plant: multiple lineages of disparate history in *Oxyria digyna*

**DOI:** 10.1002/ece3.213

**Published:** 2012-03

**Authors:** Geraldine A Allen, Kendrick L Marr, Laurie J McCormick, Richard J Hebda

**Affiliations:** 1 Department of Biology, University of Victoria,Victoria, B.C., Canada V8W 3N5; 2 Royal British Columbia Museum,675 Belleville St., Victoria, B.C. Canada V8W 9W2

**Keywords:** Arctic-alpine plants, cpDNA, phylogeography, Pleistocene glaciations, refugia

## Abstract

The ranges of arctic–alpine species have shifted extensively with Pleistocene climate changes and glaciations. Using sequence data from the *trnH*-*psbA* and *trnT-trnL* chloroplast DNA spacer regions, we investigated the phylogeography of the widespread, ancient (>3 million years) arctic–alpine plant *Oxyria digyna* (Polygonaceae). We identified 45 haplotypes and six highly divergent major lineages; estimated ages of these lineages (time to most recent common ancestor, T_MRCA_) ranged from ∼0.5 to 2.5 million years. One lineage is widespread in the arctic, a second is restricted to the southern Rocky Mountains of the western United States, and a third was found only in the Himalayan and Altai regions of Asia. Three other lineages are widespread in western North America, where they overlap extensively. The high genetic diversity and the presence of divergent major cpDNA lineages within *Oxyria digyna* reflect its age and suggest that it was widespread during much of its history. The distributions of individual lineages indicate repeated spread of *Oxyria digyna* through North America over multiple glacial cycles. During the Last Glacial Maximum it persisted in multiple refugia in western North America, including Beringia, south of the continental ice, and within the northern limits of the Cordilleran ice sheet. Our data contribute to a growing body of evidence that arctic–alpine species have migrated from different source regions over multiple glacial cycles and that cryptic refugia contributed to persistence through the Last Glacial Maximum.

## Introduction

Changing climates during the last 2 million years have profoundly influenced the distribution and abundance of temperate, boreal, and arctic species. Many species have undergone major range shifts in response to changing sea levels or glacial advances and retreats. During the Last Glacial Maximum (LGM) approximately 18,000 years ago, the Fenno-Scandian Ice Sheet in northern Europe and the Laurentide and Cordilleran Ice Sheets in North America extended over large areas of these continents (Ehlers and Gibbard 2007). Many tundra plants nevertheless have broad present-day distributions in both arctic and alpine environments, a pattern which may reflect repeated cycles of expansion, contraction, and migration over several glacial cycles. Molecular studies in combination with fossil evidence have shown diverse phylogeographic patterns among these widespread species, demonstrating how they came to occupy their present ranges and where refugia occurred (Brochmann et al. 2003; Brochmann and Brysting 2008). Both arctic refugia and southern mountain ranges have been suggested as source regions for arctic plants (Murray 1995; Brochmann and Brysting 2008). However, the extent and timelines of contributions to the arctic flora from these different source areas are still not well understood.

Beringia (Yukon to eastern Siberia) provided a large high-latitude ice-free area during the Pleistocene and has long been proposed as a major refugium for many arctic–alpine plant species during glacial periods (Hultén 1937). This hypothesis is well supported by molecular evidence (e.g., Tremblay and Schoen 1999; Abbott et al. 2000; Abbott and Comes 2003; Alsos et al. 2005; Eidesen et al. 2007; Skrede et al. 2009). Although Hultén (1937) considered Beringia the most important refugial region, he suggested that the broad distributions of many tundra species implied dispersal from additional refugia. Many arctic–alpine species persisted in refugia south of continental ice in both Europe and North America (Abbott and Brochmann 2003; Brochmann et al. 2003; Birks 2008; Brochmann and Brysting 2008); examples are *Arabis alpina* (Koch et al. 2006) and *Dryas octopetala* (Skrede et al. 2006). Additional refugia may also have existed along glacial margins, in areas such as coastal eastern Greenland (Funder 1979) and coastal British Columbia (Heusser 1989; Ogilvie 1989; Hebda et al. 1997; King et al. 2009). Recent genetic evidence has indicated survival of tundra plant and animal species in unexpected refugia within the overall margins of continental ice sheets of the Late Pleistocene, both in Europe (Westergaard et al. 2011) and in western North America (Loehr et al. 2006; Marr et al. 2008; Shafer et al. 2010a). For tundra plant species, refugia have been suggested in northwestern Europe (Westergaard et al. 2011), northeastern Canada (Tremblay and Schoen 1999), northwestern Canada (Eriksen and Töpel 2006), and northern interior British Columbia (Marr et al. 2008), but further work is needed to determine their overall importance.

Genetic evidence of rapid spread has been found in some arctic–alpine plants. For example, the ranges of *Vaccinium uliginosum* (Alsos et al. 2005; Eidesen et al. 2007) and *Rubus chamaemorus* (Ehrich et al. 2008) expanded rapidly following the LGM. Long-distance dispersal and colonization is supported by molecular evidence for a number of arctic species (Brochmann and Brysting 2008), and is the most plausible explanation for current genetic characteristics of many plants found on the remote archipelago of Svalbard (Alsos et al. 2007). However, the potential for rapid migration does not preclude the possibility of survival in multiple locations, including cryptic refugia previously unknown from fossil or palynological evidence (Stewart and Lister 2001; Provan and Bennett 2008). For example, molecular evidence suggests that some European tree species had a more complex glacial history than previously thought, including survival through the LGM in unexpected locations (Birks and Willis 2008). Tundra plants also had complex responses to past climate change (Schönswetter et al. 2005), and may have spread multiple times from steppe or montane habitats (Tkach et al. 2008). An understanding of how dispersal and refugia shaped their present distributions can provide keys to assessing their potential responses to future climate change.

**Figure 1:**
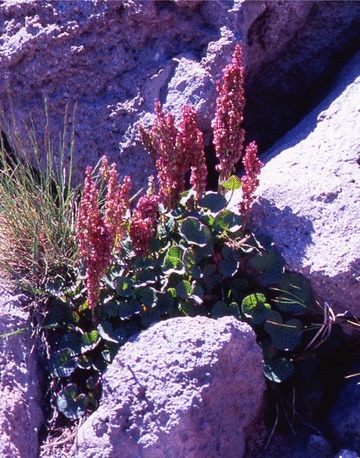
*Oxyria digyna*, Mt. Lassen, California.

**Figure 2:**
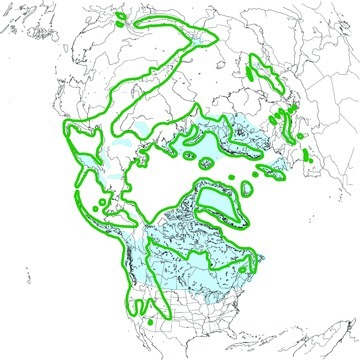
Worldwide geographic distribution of *Oxyria digyna* (green outline) and maximum extent of ice sheets during the Last Glacial Maximum (blue shading). Range map is redrawn from Hultén (1971) and glacial boundaries from Ehlers and Gibbard (2007).

*Oxyria digyna* Hill is a widespread arctic–alpine perennial herb (Fig. 1) that occurs in tundra habitats over much of the northern hemisphere (Fig. 2), including nearly all of arctic North America and Eurasia and mountain ranges of southern Russia, the Himalayan region, southern Europe, and western North America (Hultén 1971). Its fossil record extends back to the late Tertiary, with macrofossils dated at ∼3 million years reported from arctic Canada (Ellesmere, Meighen, and Prince Patrick Islands, Beaufort Formation; Mathews and Ovenden 1990). It grows in a variety of mesic tundra environments, including scree with little or no soil development. The flowers of *Oxyria digyna* are inconspicuous and wind-pollinated. The winged seeds are dispersed moderately well by wind (Tackenberg and Stöcklin 2008) and the species establishes effectively on glacial forelands (Whittaker 1993; Stöcklin and Bäumler 1996). Arctic and alpine populations show ecotypic differences in flower and rhizome production and in growth responses to temperature and day length (Mooney and Billings 1961; Heide 2005). *Oxyria digyna* is taxonomically distinct, with no closely related taxa, and is generally treated as a single well-defined species. *Oxyria digyna* is largely diploid (2*n*= 14), with a few hexaploids (2*n*= 42) reported from Russia (Fedorov 1974; Goldblatt and Johnson 1979–2010).

The history of the tundra floras of the northern hemisphere has been much more extensively investigated in Europe than in North America. In particular, phylogeographic studies of arctic–alpine plants with a focus on western North America are still relatively few (Jorgensen et al. 2003; Bain and Golden 2005), and the contributions of refugia hypothesized for this region (especially cryptic refugia) are not well understood. In a previous study (Marr et al. 2008), we used restriction fragment analysis to identify patterns of genetic diversity in western North American populations of *Oxyria digyna* in relation to recent glacial history and the locations of refugia. Here we used cpDNA sequence data to reconstruct the broader northern hemisphere phylogeographic history of *Oxyria digyna*, taking advantage of the much greater power of sequence data to resolve phylogenetic relationships among intraspecific lineages and reveal their early history. We used a combination of analytic approaches together with increased haplotype resolution and greater sampling around the northern hemisphere to address the following questions: (1) what is the geographic structure of genetic variation in *Oxyria digyna* and how does it compare with other arctic species? (2) How did the species and its major lineages diversify over time and disperse into their present geographic range? (3) What was the contribution of different refugia to the persistence of different lineages in western North America during the LGM?

## Methods

### Sampling

We sequenced 181 individuals of *Oxyria digyna* from 140 localities in North America, Europe, and Asia (Table 1). Sample localities were concentrated in western North America, but we obtained samples from much of the geographic range. We usually sampled one plant per population, but included 1–2 additional individuals in some populations from a previous study that used different methods (Marr et al. 2008). We also sampled four outgroup taxa: *Oxyria sinensis* Hemsl., *Rheum palmatum* L., *Rumex lapponicus* Czernov, and *Rumex paucifolius* Nutt. (Table 1).

**Table 1:** Collection locality information for all samples and combined *trnH-psbA* and *trnT-trnL* haplotypes for *Oxyria digyna*. Localities are arranged by continent and geographic region, and within these in order of decreasing latitude.

Country, locality	Population ID	Latitude	Longitude	Collector	Year	1Clade and haplotype
*Oxyria digyna*						
Europe and Asia						
China, Yunnan, Gaoligong Shan	YN	25° 13' N	98° 28' E	P. Fritsch	2006	F-42
Greenland, Johannes V. Jensens Land	GN	83° 24' N	29° 39' W	D. Mazzuchi	2006	A-7
Greenland, Kap K0benhavn	KAP	82° 24' N	22° 12' W	D. Mazzuchi	2006	A-7
Greenland, Zackenberg Station	ZAC	74° 28' N	20° 34' W	K. Westergaard	2007	A-7
Greenland, Osterdalen Valley	OST	69° 15' N	53° 31' W	K. Westergaard	2006	A-14
Greenland, Narsaq	NAR	60° 57' N	46° 03' W	K. Westergaard	2006	A-14
Iceland, Skalafellsjokull	SKA	64° 13' N	15° 44' W	M. Reynolds	2011	A-14
Nepal, Annapurna Sanctuary	A	28° 32' N	83° 55' E	W. Mackenzie	2005	F-41
Nepal, Khumbu Himal, Mt. Everest National Park	NEP	27° 55' N	86° 45' E	A. C. Byers	1984	F-43
Norway, Endalen Valley, Svalbard	END	78° 11' N	15° 46' E	K. Westergaard	2006	A-14
Norway, Stranda, Mt. Dalsnibba	NO	62° 03' N	7° 15' E	O. Finch	2003	A-14 (2)
Russia, Altai Republic, Rudnik Mine/Korumduairya Creek	RUD	50° 20' N	87° 45' E	K. L. Marr, R. J. Hebda	2010	F-45 (2)
Russia, Altai Republic, Karakjel Lake/Karasu Creek	KRK	50° 02' N	88° 02' E	K. L. Marr, R. J. Hebda	2010	F-44
Russia, Altai Republic, Talaya Pass	TAL	50° 00' N	89° 16' E	K. L. Marr, R. J. Hebda	2010	F-45
Russia, Kamchatka, Chazma River	KM	58° 01' N	158° 39' E	W. Merilees	2004	A-4
Russia, Magadan Oblast, near Pobieda	POB	64° 28' N	144° 55' E	K. L. Marr, R. J. Hebda	2011	A-l
Russia, Magadan Oblast, divide between Indygirka and Kolyma Rivers	IKP	63° 27' N	146° 27' E	K. L. Marr, R. J. Hebda	2011	A-l
Russia, Magadan Oblast, Burkhalinsky Pass	BUR	62° 42' N	148° 48' E	K. L. Marr, R. J. Hebda	2011	A-l
Russia, Magadan Oblast, Talaya Pass	TAY	61° 10' N	152° 00' E	K. L. Marr, R. J. Hebda	2011	A-l
Russia, Magadan Oblast, Armansky Pass	ARM	59° 40' N	150° 37' E	K. L. Marr, R. J. Hebda	2011	A-8
Russia, Sakha Republic, near Ust Nera	UST	64° 29' N	143° 15' E	K. L. Marr, R. J. Hebda	2011	A-l
Russia, Wrangel Island	WRA	71° 15' N	179° 40' W	A. Benedict	2009	A-9
Russia, Yamalo-Nenetsky Autonomous	YA	65° 27' N	66° 50' E	I. G. Alsos	2004	A-12 (2)
Region, Chernaya Mts.						
North America						
Canada, Nunavut, Alexandra Fjord, Ellesmere	AF	78° 58' N	75° 45' W	H. Guest	2003	A-6
Canada, Nunavut, Ellef Ringnes Island	RG	78° 47' N	103° 32' W	M. Reynolds	2005	A-7
Canada, Nunavut, Pond Inlet	PON	72° 45' N	77° 35' W	P. Smith	2011	A-7
Canada, Nunavut, Roche Bay	RY	68° 29' N	82° 39' W	B. Apland	2006	A-7
Canada, Nunavut, High Lake Camp	HL	67° 23' N	110° 51' W	B. Apland	2006	A-7
Canada, Nunavut, Apex, Baffin Island	BN	63° 44' N	68° 26' W	L. Gillespie	2004	A-7
Canada, NWT, Prince Patrick Island	UL	76° 14' N	119° 20' W	M. Reynolds	2005	A-7
Canada, NWT, Banks Island, DeSalis Bay	DS	71° 27' N	121° 52' W	L. Gillespie	2004	A-7
Canada, NWT, Palmer Lake Site 9	PLK	64° 32’ N	129° 34' W	A. Veitch	2007	A-6
Canada, NWT, Dechen la' North Canol Road	DEC	63° 24' N	129° 36' W	B. Krieckhaus	2007	A-5
Canada, YT, Shingle Point	SH	69° 59' N	137° 25' W	B. Bennett	2005	A-8
Canada, YT, Ivvavik National Park	IV	69° 37' N	140° 50' W	B. Bennett	2005	A-8
Canada, YT, Upper Fish Creek, Richardson Mts.	UF	67° 54' N	136° 33' W	B. Bennett	2006	A-8
Canada, YT, Mt. Klotz camp	KLO	65° 22' N	140° 11' W	B. Bennett	2007	A-6
Canada, YT, Ogilvie Mts. (site 3)	G3	64° 47' N	137° 43' W	H. Guest	2005	A-3 (2)
Canada, YT, Bonnet Plume, Gillespie Lake	BT	64° 44' N	133° 59' W	B. Bennett	2005	A-l
Canada, YT, Tombstone Territorial Park, North Fork Pass	TB	64° 35' N	138° 17' W	K. L. Marr	2004	A-6, A-8,
Canada, YT, MacMillan Pass	MP	63° 17' N	130° 09' W	B. Apland	2006	A-3
Canada, YT, Asi Keyi Special Management Area	AK	61° 34' N	140° 50' W	B. Bennett	2004	A-6
Canada, YT, Outpost Mt.	OM	60° 59' N	138° 25' W	H. Guest	2004	A-6
Canada, YT, Kotaneelee Range	KO	60° 13' N	124° 07' W	B. Bennett	2004	A-l
Canada, BC, Tagish Highland, TeePee Mt.	TEE	59° 44' N	134° 40' W	K. L. Marr, R. J. Hebda	2008	A-2
Canada, BC, Liard Plateau, Caribou Mts.	CRM	59° 43' N	125° 28' W	K. L. Marr, R. J. Hebda	2007	A-l
Canada, BC, Ruby Mt.	RB	59° 42' N	133° 22' W	K. L. Marr	2004	A-6, B-15
Canada, BC, Chuck Creek	CC	59° 42' N	136° 36' W	H. Guest	2004	A-ll
Canada, BC, Horseranch Range	HR	59° 28' N	128° 56' W	K. L. Marr, R. J. Hebda	2004	A-6
Canada, BC, Cassiar Mts., “Dakota” Lake	DL	59° 25' N	130° 13' W	K. L. Marr, R. J. Hebda	2004	A-l, A-10, B-15
Canada, BC, Kawdy Plateau, High Tuya Lake	HT	59° 15' N	130° 31' W	K. L. Marr, R. J. Hebda	2004	A-l, B-15
Canada, BC, Muskwa Ranges, Terminal Range	TER	59° 02' N	125° 55' W	K. L. Marr, R. J. Hebda	2007	A-l
Canada, BC, Nimbus Mt.	NIM	59° 02' N	132° 30' W	K. L. Marr, R. J. Hebda	2008	B-15
Canada, BC, Muskwa Ranges, Nonda Creek	N	59° 00' N	125° 30' W	K. L. Marr	2003	A-1, B-18 (2)
Canada, BC, Stikine Ranges, Four Mile Creek	4MC	58° 51' N	129° 31' W	K. L. Marr, R. J. Hebda	2007	A-6
Canada, BC, French Range, Rath Mt.	RTM	58°45' N	130° 25' W	K. L. Marr, R. J. Hebda	2007	A-6
Canada, BC, Stikine Ranges, Little Blue Sheep Lake	LBS	58° 44' N	128° 15' W	K. L. Marr, R. J. Hebda	2007	A-6
Canada, BC, Dome Mt.	DM	58°31' N	129° 35' W	K. L. Marr, R. J. Hebda	2004	A-6 (2)
Canada, BC, Snow Peak	SP	58° 20' N	130° 23' W	W. Mackenzie	2003	B-15
Canada, BC, Three Sisters Range	SI	58° 20' N	129° 45' W	W. Mackenzie	2003	A-6
Canada, BC, Stikine Ranges, Shea Mt.	SHM	58° 19' N	128° 57' W	K. L. Marr, R. J. Hebda	2007	A-6
Canada, BC, mountains south of Pitman River	PIT	57° 53' N	127° 54' W	K. L. Marr, R. J. Hebda	2009	A-6
Canada, BC, Thudaka Range	THU	57° 48' N	126° 37' W	K. L. Marr, R. J. Hebda	2009	B-15
Canada, BC, Spatsizi Provincial Park, Airplane Creek	SZ	57° 41' N	128° 52' W	R. J. Hebda	2005	A-6, B-15
Canada, BC, Mt. Edziza	E	57° 38' N	130° 40' W	C. Howers	2005	B-15 (2), B-17
Canada, BC, Moosehorn Lake	MOO	57° 35' N	127° 13' W	K. L. Marr, R. J. Hebda	2009	B-15
Canada, BC, Brothers Lake	BRO	57° 12' N	127° 26' W	K. L. Marr, R. J. Hebda	2009	E-34
Canada, BC, Kemess North	K	57° 04' N	126° 45' W	J. Pojar, W. Mackenzie	2003	A-l
Canada, BC, Wrede Range	WRE	56° 38' N	126° 12' W	K. L. Marr, R. J. Hebda	2009	B-15
Canada, BC, Oweegee Range	OW	56° 34' N	129° 28' W	K. L. Marr, R. J. Hebda	2006	B-17
Canada, BC, Chase Mt.	C	56° 33' N	125° 16' W	K. L. Marr, R. J. Hebda	2003	B-15
Canada, BC, Mt. Henri	MT	56° 29' N	124° 45' W	K. L. Marr, R. J. Hebda	2003	E-34
Canada, BC, Ludington Peak	LP	56° 28' N	123° 23' W	K. L. Marr, R. J. Hebda	2003	B-18
Canada, BC, Needham Creek	NE	56° 24' N	123° 31' W	K. L. Marr, R. J. Hebda	2003	A-l, B-15, B-18
Canada, BC, Hanna Ridge	HN	56° 14' N	129° 26' W	K. L. Marr, R. J. Hebda	2006	B-17
Canada, BC, Mt. Tommy Jack	TO	56°03' N	127° 46' W	K. L. Marr, R. J. Hebda	2006	E-34
Canada, BC, Morphee Mt.	MR	55°26' N	123° 02' W	K. L. Marr, R. J. Hebda	2003	E-40
Canada, BC, Murray Mt.	M	55° 24' N	122° 37' W	K. L. Marr, R. J. Hebda	2003	E-39
Canada, BC, Mt. Crum, “Holzworth Meadows”	H	55° 01' N	121° 31' W	K. L. Marr, R. J. Hebda	2003	E-40
Canada, BC, Babine Mts.	BA	55° 00' N	126° 56' W	K. L. Marr, R. J. Hebda	2002	E-34 (2)
Canada, BC, Nass Ranges, Mt. Couture	CU	54° 54' N	128° 42' W	K. L. Marr, R. J. Hebda	2006	E-34
Canada, BC, Sleeping Beauty Mt.	SB	54° 34' N	128° 52' W	M. Cheney	2003	B-17 (2)
Canada, BC, Mt. Thornhill	TL	54° 31' N	128° 27' W	G. Allen	2004	B-17, E-34
Canada, BC, Nadina Mt.	NA	54° 06' N	126° 52' W	K. L. Marr, R. J. Hebda	2002	E-34 (2)
Canada, BC, Tweedsmuir Peak	TW	53° 39' N	126° 29' W	K. L. Marr, R. J. Hebda	2002	D-32 (2)
Canada, BC, Canoe Mt.	CA	52° 43' N	119° 11' W	K. L. Marr	2002	E-35 (2)
Canada, BC, Mt. De La Touche.	LT	52° 42' N	132° 02' W	G. W. Douglas	2003	B-17
Canada, BC, Itcha Ilgachuz	CH	52° 42' N	124° 51' W	R. Coffey	2003	E-35
Canada, BC, Mackenzie Mt.	MK	52° 35' N	126° 09' W	W. Mackenzie	2003	E-35
Canada, BC, Tweedsmuir Park, Crystal Lake trail	CL	52° 32' N	125° 49' W	R. Coffey	2003	D-32, E-35
Canada, BC, Caput Mt.	CAP	52° 25' N	120° 36' W	F. Osorio	2006	E-40
Canada, BC, Wells-Gray Provincial Park, Trophy Mt.	TR	51° 47' N	119° 55' W	K. L. Marr	2002	E-40
Canada, BC, Niut Range	NT	51° 43' N	124° 39' W	W. Mackenzie	2003	E-35 (2)
Canada, BC, Brisco Range, Kindersley Pass	KIN	50° 42' N	116° 00' W	K. L. Marr	2007	E-34
Canada, BC, Bugaboo Pass	BO	50°41' N	116° 45' W	O. McDadi	2004	E-34, E-40
Canada, BC, Paradise Mine	PA	50° 29' N	116° 18' W	K. L. Marr	2002	D-32, E-34
Canada, BC, Vancouver Island, Merry Widow Mt.	MW	50° 21' N	127° 18' W	K. L. Marr, R. J. Hebda	2005	E-34
Canada, BC, Kootenay Lake	KOO	50° 18' N	117° 08' W	W. Mackenzie	2007	E-39
Canada, BC, Whistler Mt.	WH	50° 04' N	122° 57' W	K. L. Marr	2003	E-35
Canada, BC, Manning Park	MA	49° 6' N	120° 46' W	K. L. Marr	2002	E-40 (2)
Canada, BC, Gimli Peak	GI	49° 46' N	117° 39' W	H. Guest	2006	E-35
Canada, BC, Vancouver Island, above Circlet Lake	FP	49° 41' N	125° 22' W	K. L. Marr	2003	E-34
Canada, BC, Fernie, Three Sisters trail	3S	49° 35' N	115° 08' W	K. L. Marr	2002	E-35 (2)
Canada, BC, Vancouver Island, Mt. Arrowsmith	AR	49° 14' N	124° 36' W	K. L. Marr	2004	D-32
Canada, BC, Silver Tip Mt.	SM	49° 10' N	121° 13' W	G. W. Douglas	2003	E-35
Canada, BC, Kishinena Range	KR	49° 02' N	114° 16' W	W. Mackenzie	2005	E-35
Canada, AB, Pipestone R. Trail	PP	51° 35' N	116° 09' W	S. White	2003	E-35
Canada, AB, Lower Fish Lake	LF	51° 31' N	ii6° irw	S. White	2003	E-35
USA, AK, Beaufort Sea coast, Camden Bay	BFS	69° 58' N	144° 46' W	J. Jorgensen	2007	A-13
USA, AK, Anaktuvuk Pass, Alaska	ANA	68° 08' N	151° 36' W	B. Krieckhaus	2008	A-6
USA, AK, Dot Lake	DK	63°39' N	144° 37' W	H. Guest	2005	A-10
USA, AK, Richardson Highway	RH	63°21' N	145° 42' W	H. Guest	2005	A-l (2)
USA, AK, Caribou Creek	CR	62° 37' N	143° 28' W	H. Guest	2005	A-10
USA, AK, Chitina Rd.	CM	61°34' N	144° 36' W	H. Guest	2005	A-3 (2)
USA, AK, Crow Pass	CW	61° 02' N	149° 07' W	H. Guest	2005	A-l
USA, AK, Bering Sea, St. Matthew Island	HSM	60° 33' N	172° 42' W	CAN 587881	1944	A-l
USA, AK, Carbon Mt.	CO	60° 27' N	143° 53' W	A Batten	2005	B-18, B-19
USA, AK, Kenai Fjords	KF	59° 27' N	149° 57' W	M. Carlson	2005	B-15
USA, AK, Chichagof Island, Hoonah Ridge	CF	57° 42' N	135° 02' W	B. Krieckhaus	2006	B-15 (2)
USA, AK, Kodiak Island	KD	57° 16' N	154° 12' W	C. Parker	2005	B-15
USA, AK, Unalaska Island, Pyramid Peak	UNF	53° 51' N	166° 32' W	B. Krieckhaus	2007	B-16
USA, WA, Winchester Mt. Lookout	WIN	48° 57' N	121° 39' W	K. L. Marr	2007	E-34
USA, WA, Mt. Baker, Bagley Lakes Trail	BAG	48° 51' N	121° 41' W	K. L. Marr	2007	E-34
USA, WA, Hart's Pass	HP	48° 44' N	120° 40' W	K. L. Marr	2003	E-35, E-40
USA, WA, Olympic Mts., Obstruction Point	OP	47° 55' N	123° 23' W	K. L. Marr	2003	B-21, D-32
USA, WA, Chinook Pass	1R	46° 52' N	121° 31' W	G. Allen, K. L. Marr	2004	E-39
USA, MT, Anaconda Pintlar Range, Seymour Lake	SY	46° 03' N	113° 58' W	H. Guest	2006	E-35
USA, MT, Absaroka Mts.	AB	45° 40' N	110° 34' W	P. Lesica	2004	B-23, B-25
USA, MT, Hyalite Creek	HY	45° 26' N	110° 57' W	K. L. Marr	2005	D-30, E-37
USA, ID, Heaven's Gate	HG	45° 22' N	116° 30' W	G. Allen, K. L. Marr	2004	B-24
USA, ID, Seven Devils Mts.	SD	45°21' N	116° 31' W	G. Allen, K. L. Marr	2004	E-36
USA, OR, Mt. Hood, Elliott Glacier	EG	45° 24' N	121° 40' W	G. Allen, K. L. Marr	2004	B-20 (2)
USA, OR, Three-Fingered Jack	TJ	44° 24' N	121° 48' W	G. Allen, J. Antos	2004	E-39
USA, OR, Strawberry Mts.	SW	44° 18' N	118° 41' W	G. Allen, K. L. Marr	2004	B-22, E-38
USA, OR, Mt. Ashland	AS	42° 05' N	122° 43' W	G. Allen, J. Antos	2004	D-31
USA, WY, Wind River Range, Haystack Mt.	HM	42° 44' N	109° 10' W	J. Dunn	2006	C-28
USA, WY, Medicine Bow Mts.	MD	41° 21’ N	106° 20’ W	H. Guest	2006	C-27
USA, CO, Forest Lakes	FL	39° 55' N	105° 40' W	K. L. Marr	2003	C-26, C-29
USA, CA, Mt. Lassen	L	40° 29' N	121° 31' W	G. Allen, J. Antos	2004	D-31
USA, CA, sw of Bishop, Paiute Pass Trail	PU	37° 14' N	118° 39' N	H. Guest	2006	E-35
Outgroups						
*Oxyria sinensis*						
China, Yunnan, Deqing		57° 59' N	98° 27' E	L. Q. Jun		2006
*Rheum palmatum*						
Canada, Jardin botanique de Montréal (cultivated)		—	—	G. Allen		2008
*Rumex lapponicus*						
Canada, BC, Anthony Creek		56° 49' N	128° 35' E	K. L. Marr, R. J. Hebda		2005
*Rumex paucifolius*						
USA, Wyoming, Wind River Mts., Green River Lake		43° 17' N	109° 50' E	G. Allen, J. Antos		2005

1 Parenthese indicate multiple samples from the same locality.

### DNA extraction, amplification, and sequencing

DNA was extracted from silica-dried leaves (or in a few cases, pressed specimens) from 1–3 plants per population, using either a modified CTAB method (Doyle and Doyle 1987) or DNeasy Plant Mini Kits (Qiagen, Valencia, CA). We amplified and sequenced two cpDNA noncoding regions, the *trnH*-*psbA* and *trnT-trnL* spacers, in all samples including outgroups. We also sequenced a third region, the *ndhJ-trnF* spacer, in a subset of 37 *Oxyria digyna* samples and in the outgroups. For the *trnH*-*psbA* region we used the primers psbA (Sang et al. 1997) and trnH (Tate and Simpson 2003). For the *trnT-trnL* region we used forward primers Tab A (Taberlet et al. 1991) or trnA2 (Cronn et al. 2002) with reverse primers Tab B (Taberlet et al. 1991) or Oxy88 (Marr et al. 2008). For the *ndhJ-trnF* region we used forward primer ndhJ (Shaw et al. 2007) and reverse primer trnF (Dumolin–Lapegue et al. 1997).

PCR was performed in 50 µL volumes with the following reagents: 5 µL template DNA (1:10 dilutions), 5 µL 10× PCR Buffer, 5 µL 2 mM dNTPs, 0.25 µL Taq DNA polymerase (New England Biolabs, Ipswich, MA), and 2.5 µL of 5 µM of each primer. All PCR reactions were carried out using Techne TC-312 thermal cyclers. PCR parameters for amplification of the *trnH*-*psbA* and *trnT-trnL* regions were 3 min at 94°C, 30 cycles of 94°C for 30 s, 57°C for 1 min, and 72°C for 1 min, with a final 10 min at 72°C. Parameters for the *ndhJ-trnF* region were 5 min at 80°C, 30 cycles of 95°C for 1 min, 50°C for 1 min, and 65°C for 4 min, with a final 5 min at 65°C. In preparation for sequencing, PCR products were purified using the QIAquick PCR Purification Kit (Qiagen, Valencia, CA). All samples were sequenced in both primer directions using the amplifying primers. DNA sequencing was carried out by Macrogen Inc. (Seoul, Korea), using ABI 3700 or ABI 3730XL PRISM capillary sequencers (Applied Biosystems, Foster City, CA). To minimize PCR errors, all rare sequence variants were reamplified and resequenced.

### Data analyses

Sequences for each DNA region were aligned using ClustalX (Thompson et al. 1997) and Jalview (Waterhouse et al. 2009), and manually adjusted to minimize the number of length variations. Three of the four outgroup taxa were highly divergent from *Oxyria digyna* in all sequenced regions and were not used further. *Oxyria sinensis* aligned satisfactorily with *Oxyria digyna* for the *ndhJ-trnF* region only. We performed maximum parsimony (MP) and maximum likelihood (ML) analyses of the *ndhJ-trnF* region with *Oxyria sinensis* as outgroup; the resulting trees showed little resolution of relationships among *Oxyria digyna* haplotypes and are not reported here. All reported analyses are based on the *trnH*-*psbA* and *trnT-trnL* spacer regions.

Haplotype networks for the *trnH*-*psbA* and *trnT-trnL* noncoding regions, separately and in combination, were constructed using the statistical parsimony software TCS v. 1.21 (Clement et al. 2000). An ILD test (Michewich and Farris 1981), as implemented in PAUP 4.0b10, was carried out using 500 random replicates to check for partition heterogeneity of the two regions. Indels were treated as unit characters and included in all analyses. To obtain estimates of support for branches of the combined network, we performed MP and Bayesian phylogenetic analyses on a data set consisting of one sequence for each combined haplotype (with indels coded as 0/1 characters in a separate data partition). MP analysis was performed with PAUP 4.0 version b10 (Swofford 1998), with clade support estimated from 500 bootstrap replicates using a heuristic search with TBR branch swapping. Bayesian analysis was carried out with MrBayes v. 3.1.2 (Huelsenbeck and Ronquist 2001) under the assumption of a strict molecular clock, using a GTR substitution model as determined with jModelTest 0.1.1 (Guindon and Gascuel 2003; Posada 2008). We executed two sets of four Markov chain Monte Carlo (MCMC) runs for 2 million generations, sampling every 100th generation, with burnin of 25%.

We estimated time to most recent common ancestor (T_MRCA_) for major lineages (with ≥5 samples) identified in the phylogenetic analyses, using BEAST software (Drummond and Rambaut 2007). Analyses were based on the complete data set of 181 sequences and all characters except indels (which were excluded because they are difficult to incorporate into coalescent analyses). Two independent analyses were each run for 10^8^ generations under a GTR substitution model, selected using jModeltest 0.1.1. Analyses were combined and evaluated using Tracer 1.5 (Rambaut and Drummond 2007). In preliminary analyses assuming a relaxed (uncorrelated lognormal) molecular clock or exponential population growth, marginal posterior distributions of (respectively) clock rate variation and population growth rate included zero; we therefore assumed a strict molecular clock and constant population size for all subsequent runs. In order to estimate T_MRCA_ in years, replicate analyses were carried out with calibration based on either fossil evidence or a published mutation rate. For fossil calibration we assigned an age of 3.0–3.4 million years, the estimated age of the oldest known *Oxyria digyna* macrofossil (Matthews and Ovenden 1990), to the root of the tree (using a normally distributed prior of 3.2 ± 0.14 million years, to give a 95% confidence interval of approximately the above range). For calibration from mutation rate, we used a mean substitution rate of ∼1.52 ± 0.06 × 10^–9^ substitutions per site per year as estimated for chloroplast noncoding regions by Yamane et al. (2006).

We investigated possible expansion events in the histories of major lineages of *Oxyria digyna* by performing mismatch analyses using Arlequin v. 3.5 (Excoffier et al. 2005) on all lineages with five or more samples (Clades A, A + C, B, D, E, and F). Mismatch analyses test the hypothesis that a mismatch distribution (the distribution of all possible pairwise differences for a group of sequences) approximates the unimodal shape predicted for a population or lineage that has undergone a demographic or spatial expansion event (Excoffier 2004); the mean number of pairwise differences can be used to infer time since an expansion event.

In western North America, the geographic area for which we had the highest sampling density, we assessed spatial patterns of genetic diversity for five different geographic regions. These were delineated to approximately reflect differences in tundra environment (high arctic to southern alpine) and glacial history (glaciated or not), as follows: (1) Alaska and the Yukon; (2) Northwest Territory and Nunavut; (3) northern British Columbia (latitudes 54.5–60.0°N); (4) southern British Columbia (latitudes 49.0°–54.5°N); and (5) continental western USA (south of 49.0°N). We tabulated overall numbers of haplotypes and numbers of haplotypes unique to a region (across all clades and within major clades) and calculated genetic diversity, Ĥ (the probability that two randomly chosen haplotypes in a sample are different) and molecular diversity, π (the mean number of pairwise differences between haplotypes in a sample), both within clades and overall clades for each region. Both diversity measures are sensitive to numbers and relative abundances of haplotypes; π also reflects divergence among haplotypes. All genetic diversity measures were calculated using Arlequin v. 3.5 (Excoffier et al. 2005).

## Results

### Sequence variation

Total aligned length of the *trnH*-*psbA* region was 315 bp, with 24 variable characters (18 substitutions, five indels of 5 to 8 bp in length, and one single-base T repeat). Total aligned length of the *trnT-trnL* region was 910 bp, with 68 variable characters used for analyses (41 substitutions and 27 indels of 3 to 19 bp); five additional 1- or 2-bp repeats could not be aligned unambiguously and were omitted from all analyses. There was no evidence for heterogeneity of the two regions (ILD test in PAUP*, *P*= 0.11) and all inferences are based on the combined two-region data set with an aligned length of 1225 bp. Sequences representing all distinct variants found in this study are deposited in GenBank; accession numbers are JQ080918-JQ080962 for the *trnH*-*psbA* region and JQ080963-JQ081007 for the *trnT-trnL* region.

### Phylogenetic relationships among haplotypes

**Figure 3:**
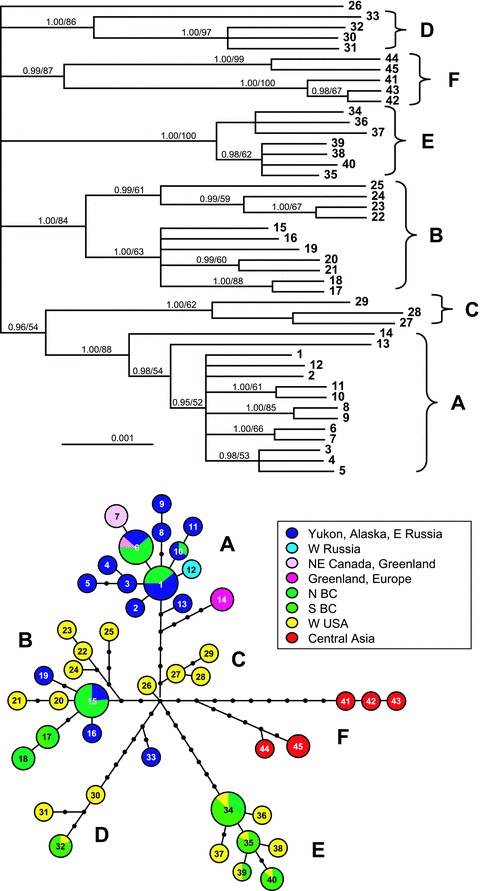
Relationships among *Oxyria digyna* haplotypes from the combined analysis of *trnH* - *psbA* and *trnT-trnL* cpDNA spacer regions. (A) Bayesian phylogenetic tree, with clade support indicated by Bayesian posterior probabilities and maximum parsimony bootstrap values. (B) TCS haplotype network. Circle diameters are proportional to the number of samples of each haplotype; connecting dots indicate inferred haplotypes not found in the data set. Colors indicate the geographic occurrences of haplotypes.

TCS analysis of the combined *trnH*-*psbA* and *trnT-trnL* data set yielded a total of 45 haplotypes (Fig. 3), which formed distinct groups corresponding with strongly supported clades (all with Bayesian posterior probabilities of 0.99–1.00) identified in the phylogenetic analyses (Fig. 3a). These included a widespread clade (A) comprising nearly all arctic samples; four clades (B to E) with variously overlapping geographic distributions in western North America; and a clade (F) found only in the mountains of central and southern Asia. A single, additional haplotype (26) was of indeterminate phylogenetic position but had greatest sequence similarity to Clade C (Fig. 3b). Haplotypes in sampled populations are indicated in Table 1.

Although each of the six major clades was strongly supported, relationships among them were mostly unresolved in the Bayesian phylogenetic analysis (Fig. 3a). Clades A and C formed a well-supported higher-level clade, A + C (with posterior probability of 0.96; Fig. 3a).

### Timelines and clade histories

Estimated T_MRCA_s for the analyzed major clades suggest that at least some of these have a very long history spanning much of the Pleistocene (Table 2). Estimated coalescence times were similar whether calibrated with fossil evidence or mutation rate. Clade F was identified as the oldest clade (mean T_MRCA_ 2.58–3.02 × 10^6^ years). The combined Clades A + C (mean T_MRCA_ 1.42–1.66 × 10^6^ years) and Clade D (mean T_MRCA_ 1.27–1.45 × 10^6^ year) appear to be substantially older than the remaining clades (A, B, and E), of which the youngest, Clade E, had a mean T_MRCA_ of 458–516 × 10^3^ years (Table 2). Data for Clade C were insufficient for analysis.

**Table 2:** Estimates of time to most recent common ancestor (T_MRCA_) in years before present for major lineages of *Oxyria digyna*, based on combined *trn* H- *psb* A and *trn* T-L cpDNA sequence data.

Lineage	T_MRCA_ (from fossil date)^1^ Mean ± SD	T_MRCA_ (from substitution rate)^2^ Mean ± SD
*O. digyna* (all extant clades combined)^3^	–	3,705,000 ± 821,000
Clade A	769,000 ± 279,000	893,000 ± 266,000
Clades A + C	1,415,000 ± 545,000	1,656,000 ± 583,000
Clade B (all)	777,000 ± 294,000	911,000 ± 288,000
Clade B (northern subclade)	475,000 ± 191,000	561,000 ± 190,000
Clade D	1,265,000 ± 518,000	1,454,000 ± 507,000
Clade E	458,000 ± 204,000	516,000 ± 199,000
Clade F	2,576,000 ± 696,000	3,024,000 ± 1,188,000

1 based on late Tertiary macrofossils of *O. digyna* (Matthews and Ovenden 1990).

2 based on substitution rate of Yamane et al. 2006) for cpDNA noncoding regions.

3 calculated from substitution rate only.

Mismatch analyses of major clades (A, A + C, B, D, E, and F) suggested spatial expansion events in the histories of all clades (*P* > 0.05 for all tests of fit to the model). Mismatch distributions were approximately unimodal for all of these clades, but with a second, small peak (suggesting the possibility of multiple expansion events) in Clade A + C and in Clade D. Estimates of τ had very broad 95% confidence limits for all clades and were insufficiently accurate for useful estimates of times since expansion; these are not reported here.

### Haplotype diversity and geographic distribution

Haplotype diversity of *Oxyria digyna* in western North America (Table 3) varied among geographic regions, both overall and within clades. Overall haplotype diversity measures Ĥ and π were high in Regions 1 (Alaska/Yukon), 3 (northern British Columbia), and 5 (continental western USA) and lowest in Region 2 (Nunavut and Northwest Territories). Haplotype diversity was low in Region 4 (southern British Columbia), in marked contrast to Region 3 (northern British Columbia). The high overall values of π in several regions, especially Regions 3 and 5, reflect the presence of haplotypes belonging to divergent major clades in this region (Table 3). Haplotypes from several clades were present in all regions except Region 2. Within individual clades, haplotype diversity varied with latitude across geographic regions, tending to be highest either in the north (Region 1) or in the south (Region 5) (Table 3). These two geographic regions also harbored unique (region-specific) haplotypes for all major clades that were present, and unique haplotypes were concentrated in these regions (Table 3).

**Table 3:** *Oxyria digyna* genetic diversity statistics by geographic region for localities in western North America, based on combined *trn* H- *psb* A and *trn* T-L cpDNA sequence data. Diversity measures for each region are given for all haplotypes combined (in bold) and for haplotypes within each major clade.

Region	Clade	No. of samples	No. of haplotypes	No. of unique haplotypes	Gene diversity, Ĥ	Molecular diversity, π
Region 1 (Alaska/Yukon)	**All**	**29**	**11**	**6**	**0.894**	**4.259**
	A	23	6	3	0.834	1.652
	B	5	4	2	0.900	2.400
	D	1	1	1	0	0
Region 2 (Nunavut/NWT)	**A (All)**	**9**	**3**	**2**	**0.556**	**1.667**
Region 3 (northern BC)	**All**	**51**	**11**	**2**	**0.842**	**6.753**
	A	21	5	2	0.638	0.895
	B	22	3	0	0.507	1.221
	E	8	3	0	0.607	1.643
Region 4 (southern BC)	**All**	**39**	**6**	**0**	**0.775**	**4.289**
	B	4	1	0	0	0
	D	5	1	0	0	0
	E	30	4	0	0.653	1.076
Region 5 (w USA s of 49°N)	**All**	**26**	**20**	**15**	**0.979**	**6.200**
	B	7	6	6	0.952	4.762
	C	3	3	3	0.909	3.000
	D	4	3	2	0.833	3.333
	E	11	7	3	0.909	1.927
	hap. 26	1	1	1	0	0

**Figure 4:**
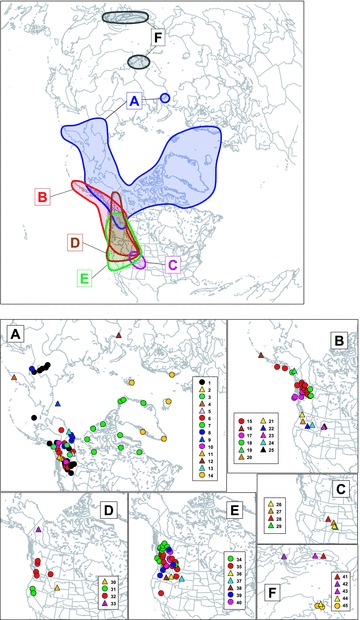
Geographic distributions of all haplotypes (Fig. 3, Table 1) from the combined analysis of *trnH* - *psbA* and *trnT-trnL* cpDNA spacer regions of 181 samples of *Oxyria digyna*. (A) Major clades. (B) Individual haplotypes within major clades (haplotype 26 is included on the Clade C map). Haplotypes occurring at multiple localities are indicated by circles and those found at only one locality by triangles. Colors are used only to differentiate haplotypes and do not carry geographic information. Haplotype numbers correspond to those in Fig. 3.

Three of the major lineages appear to have very disparate geographic distributions, with little overlap (Fig. 4a). The single Eurasian lineage, Clade F, is genetically and geographically remote from all other haplotype groups detected (although it was undoubtedly undersampled in this study). Clade C was found only in a small region of the Rocky Mountains in the western USA. The grouping of this small clade with the northern Clade A in the phylogenetic analysis suggests a formerly more contiguous distribution over the intervening region. The largely arctic Clade A is the most widely distributed lineage (Fig. 4a, b), spanning a continuous longitudinal range of at least 195° (from eastern Greenland across North America to eastern Siberia), and extending to 270° with inclusion of the Yamalo–Nenetsky region of Russia. Clade A comprises a subclade of closely related haplotypes (1 to 13) and a single divergent haplotype (14) of trans-Atlantic distribution (Greenland, Svalbard, and northern Europe). The main subclade is widely distributed, with a concentration of haplotypes in northwestern North America. Within this subclade, the earliest diverging haplotypes 1 and 13 were found from southwestern Alaska to northern British Columbia (Fig. 3b, 4b), with the highest diversity of haplotypes also in this region.

Clades B, D, and E all occur in western North America, mostly south of Alaska and the Yukon, with broad geographical overlap (Table 3, Fig. 4a, b). Their relative abundances and the distribution patterns of haplotypes within each clade suggest different histories. Clade B includes 11 haplotypes divided into two well-marked and geographically distinct subclades (Fig. 3, Fig. 4b). The predominantly northern subclade (haplotypes 15 to 21) occurs primarily from southern coastal Alaska to northern British Columbia, with a disjunct southern lineage (haplotypes 20 and 21). A second, southern subclade (haplotypes 22 to 25) is restricted to cool, damp alpine habitats in the western USA. Clade D is a sparse clade with four divergent haplotypes (30 to 33) distributed over a large geographic region in western North America (Fig. 4b). Haplotype 33 in this clade, identified from a single northern Yukon locality, is sister to a clade of the remaining three haplotypes, found further south. Clade E with seven haplotypes (34–40) is geographically well-defined, occurring from north-central British Columbia to central California. Clades B, D, and E overlap in a large contact zone extending over much of the formerly glaciated region of western North America.

## Discussion

### An old and complex history

*Oxyria digyna* is an old and morphologically distinct species (Murray 1995; Brochmann and Brysting 2008). Its diversity of chloroplast haplotypes is consistent with its long fossil history extending back to the late Tertiary (Fredskild and Røen 1982; Mathews and Ovenden 1990; West 2000; Akopian et al. 2008; Bennike et al. 2010). Chloroplast haplotype diversity in *Oxyria digyna* is greater than in most other widely distributed arctic–alpine species investigated to date, including *V. uliginosum* (Alsos et al. 2005; Eidesen et al. 2007), *A. alpina* (Koch et al. 2006), and *Juncus biglumis* (Schönswetter et al. 2007), and is comparable to genetic diversity of multispecies lineages in genera such as *Hordeum* (Jakob and Blattner 2006). Alsos et al. (2005) identified three major cpDNA lineages within the arctic shrub *V. uliginosum*, which also has a Tertiary fossil history, and concluded that the earliest divergence of these lineages probably occurred at the beginning of the Quaternary glaciations (>700,000 years). Our estimated overall T_MRCA_ for *Oxyria digyna* based on published molecular substitution rates suggests an origin as long ago as the late Tertiary (∼3.7 million years; Table 2), which is consistent with the fossil evidence. Murray (1995) suggested that *Oxyria digyna* is one of the oldest arctic–alpine species.

*Oxyria digyna* shows a striking phylogenetic split into at least six deeply divergent cpDNA lineages. Although relationships among these clades were not resolved in the phylogenetic analyses, estimated times to most recent common ancestor (T_MRCA_) for the different clades indicate that they are of substantially different ages. All of the major clades appear to be old (∼0.5–2.5 × 10^6^ years), and some lineages (clade A + C, clade D, clade F) may have originated in the early Pleistocene. Molecular evidence increasingly indicates (Brochmann and Brysting 2008) that many species in the relatively young (2–3 million year old) arctic tundra ecosystem harbor greater genetic variation than expected. However, among arctic species for which data are available, only *Saxifraga oppositifolia* appears to have as much divergence among major cpDNA lineages (Abbott et al. 2000) as does *Oxyria digyna*. The presence of divergent lineages within a species indicates not only a long history, but sufficiently large populations that genetic diversity is maintained over time. Although shifts in climate through the Pleistocene may have led to genetic bottlenecks for many arctic–alpine species, highly cold-adapted species may have maintained very large ranges that simply shifted in response to climate cycles (Brochmann and Brysting 2008). This is a likely scenario for the very widespread and cold-tolerant *Oxyria digyna*.

Pan-arctic taxa are of diverse origins. Some of the oldest species may have arisen in southern alpine habitats and others in long-persistent high-latitude regions such as Beringia (Abbott and Brochmann 2003; Brochmann and Brysting 2008). Among widely distributed species, multiple sources seem likely. Evidence concerning the geographic origins of *Oxyria digyna* is equivocal. Its nearest relatives occur in Asia; its only congener, *Oxyria sinensis,* is restricted to western China and the sister taxon of *Oxyria* is the wholly Eurasian genus *Rheum* (Frye and Kron 2003). However, the oldest known fossils of *Oxyria digyna* are in arctic Canada and in Greenland. *Oxyria digyna* may have originated in central or eastern Asia but diversified in North America early in its evolution. The high haplotype diversity of major clades in northwestern North America suggests a long history of *Oxyria digyna* in this region. The complex patterns of lineage distribution and overlap in western North America likely reflect a series of regional extinction and recolonization cycles associated with multiple climatic fluctuations and glacial episodes in the later Pleistocene.

### Histories of major lineages

The differing geographic distributions of *Oxyria digyna* lineages imply different Pleistocene histories. The western North American Clade C and the Eurasian Clade F were found only in southern alpine areas. *Oxyria digyna* is disjunct in northern and southern Russia (Hultén 1971; Fig. 2) and Clade F may represent a southern lineage, although it is probably more widespread than our sampling indicates. Hultén (1937) and others have suggested that many widely distributed arctic species migrated alternately south and north as glaciers advanced and retreated, sometimes resulting in disjunct geographic ranges with isolated southern populations.

In contrast, the widespread arctic distribution of Clade A suggests a high latitude origin. Clades A and C form a higher-level clade, which could have spread from Asia or possibly originated in western North America with subsequent divergence of Clades A and C to the north and south. The high haplotype diversity of Clade A in northwestern North America and the distribution of its ancestral haplotypes are consistent with a long presence in the Beringian region, with more recent eastward and westward spread through arctic regions. *Oxyria digyna* is moderately well dispersed by wind (Tackenberg and Stöcklin 2008) and probably was able to spread rapidly into new habitat as continental ice sheets receded and climatic conditions became more favorable. Central and eastern regions of arctic Canada and northern Greenland are occupied almost exclusively by a single derived haplotype, suggesting recent migration. The history of arctic lineages of *Oxyria digyna* in northern Asia is less clear. Fossils indicate that it occurred in northern Siberia during and preceding the LGM (Kienast et al. 2001; Lopatina and Zanina 2006), and clade A haplotypes in northwestern Siberia are closely related to those from western North America. Broad geographic regions of low haplotype diversity, with higher diversity in refugial areas such as Beringia, have been observed in other arctic plants, including *A. alpina* (Koch et al. 2006), *Ranunculus glacialis* (Schönswetter et al. 2003), and *Draba* species (Skrede et al. 2009). The single amphi-Atlantic haplotype 14 in Clade A may represent a distinct lineage that persisted through the LGM in either European or northeast North American refugia.

Three lineages of *Oxyria digyna* (Clades B, D, and E) have an unusually broad zone of overlap extending over much of British Columbia and adjacent regions. The sympatry of these divergent cpDNA lineages and the geographic distributions of haplotypes within each lineage indicate a long and complex history of *Oxyria digyna* in western North America, involving repeated expansion, migration, and local extinction. Mismatch analyses suggested spatial expansion events in the histories of all these clades, though we could not ascertain whether these events coincided with initial clade divergence or occurred subsequently. The differences in estimated age and relative abundance of these clades suggest successive episodes of migration from one or more source regions. Clade D is an old (∼1.2–1.5 × 10^6^ years), sparse clade and probably represents an early-colonizing lineage of which only remnant haplotypes now remain. Clades B and E, in contrast, are of more recent origin (∼0.4–0.9 × 10^6^ years). Clades B and D both show evidence of range fragmentation resulting from glacial advances. Clade B has a particularly complex pattern of north–south disjunctions between related haplotypes, suggesting multiple fragmentation events in its history.

### Persistence through the last glacial maximum in western North America

In species with a long history, early distributions of lineages are difficult to infer because of the strong influence of later events. Present-day spatial patterns of genetic variation are often best understood in relation to the most recent glaciation and associated climatic changes. This is true for *Oxyria digyna*, although the time since the LGM (18,000 years) represents less than 1% of the history of the species (>3 million years) and thus almost all of the observed haplotypes must predate the LGM. Most tundra and boreal species now present in western North America persisted through the last glacial maximum north or south of the continental ice sheets (Jorgensen et al. 2003; Beatty and Provan 2010); some also persisted in refugia between these limits (Loehr et al. 2006; King et al. 2009). *Oxyria digyna* gives evidence of persistence in all of these areas, with different refugia harboring different combinations of clades.

The ice-free area of Beringia formed a northern refugium extending over most of Alaska and the northern half of the Yukon (Fig. 2) in which Clades A and D almost certainly persisted through the LGM. Clade A is widespread in the Beringian region, which is also where its haplotype diversity is highest. Clade D includes a haplotype (33) that was found only in Beringia; the other haplotypes occur much farther south and likely persisted elsewhere. Clade B may also have persisted in Beringia or nearby cryptic refugia; it is distributed along the southern margin of Alaska including the Aleutian Islands.

Areas south of the continental ice sheets also provided extensive suitable habitat. Two separate lineages within Clade B and all haplotypes of Clade C are presently restricted to cool moist alpine habitats south of the limits of continental ice. These are probably remnants of much more extensive populations that were widespread during the LGM and subsequently became isolated into small, disjunct patches of alpine habitat. Clade E and the southern subclade of group D also occur south of the glacial boundary, but extend north into formerly glaciated regions of British Columbia. These likely persisted, at least in part, south of the continental ice sheets, migrating northward as the ice receded. Clade E occurs from the western USA north over much of British Columbia, with some haplotypes found almost entirely within the limits of the Cordilleran ice sheet but with highest diversity of haplotypes in the south. Northward movement from south of the Cordilleran ice sheet and persistence within its boundaries are both plausible for this clade.

Our evidence suggests persistence of *Oxyria digyna* within the boundaries of the Cordilleran ice sheet during the LGM. We observed marked differences in haplotype diversity between northern and southern British Columbia (Table 3, Fig. 4b), although these regions, both within the boundaries of the Cordilleran ice sheet, are thought to have had a similar glacial history over the last 25,000–30,000 years. Genetic diversities of Clades A and B were nearly as high in northern British Columbia as in adjacent unglaciated regions further north, and included haplotypes found only within the boundaries of the Cordilleran ice sheet. In contrast, within-clade genetic diversities in the southern part of the ice sheet are much lower, implying continuous ice cover at the LGM. One hypothesis to explain these differences is that the extensive tundra habitat of northern refugia facilitated more extensive postglacial migration of *Oxyria digyna* from the north. However, haplotype diversity of *Oxyria digyna* is even higher south of the Cordilleran ice sheet than north of it (Table 3), yet most of this variation is not found in southern British Columbia. Thus persistence of *Oxyria digyna* in ice-free habitats within the northern Cordilleran ice sheet, rather than differential subsequent migration, appears to be the most likely explanation for the observed pattern of haplotype diversity. Persistence in northern British Columbia during the LGM is also indicated for two vertebrates (Loehr et al. 2006, Shafer et al. 2010a), and may have been possible for other species (Shafer et al. 2010b). The occurrence of refugial habitat within the margins of continental ice sheets in Europe is similarly supported by recent molecular studies (Brochmann et al. 2003; Westergaard et al. 2011).

## Conclusions

*Oxyria digyna* is a very old species, as indicated by both fossil evidence and the cpDNA patterns reported here. It has higher genetic diversity than most arctic–alpine species studied thus far, reflecting its age, but also suggesting a widespread distribution over time with few bottlenecks. Its history rivals that of multispecies complexes, reflecting both ancient and more recent cycles of range contraction and expansion. We show that different lineages within a single species may have very different histories and dispersal patterns spanning hundreds of thousands of years. Although the geographical origin of *Oxyria digyna* is most likely in Eurasia, our evidence suggests a long and complex history of this species in western North America. Differing ages of lineages and distributions of haplotypes indicate repeated migrations over multiple glacial cycles. Our genetic evidence indicates refugia for *Oxyria digyna* not only north and south of continental ice sheets but also within the limits of the northern Cordilleran ice sheet in northern British Columbia. Thus our findings support the emerging generalization that cryptic refugia were important for plant survival and recolonization of glaciated regions, as already demonstrated for Europe (Brochmann et al. 2003; Brochmann and Brysting 2008).
